# Proteomic and Metabolomic Analysis Reveals Candidate Biomarkers and Meat Quality Differences in Divergent Climatically Adapted Sheep Breeds

**DOI:** 10.3390/foods15111962

**Published:** 2026-06-02

**Authors:** Yaling Yang, Wujun Liu, Hang Cao

**Affiliations:** Department of Animal Science, Xinjiang Agricultural University, Urumqi 830052, China; yangyaling3141@163.com (Y.Y.); lwj_ws@163.com (W.L.)

**Keywords:** Turpan Black sheep, Altay sheep, metabolomics, proteomics, extreme environment adaptation, meat quality

## Abstract

Turpan Black (TBL) and Altay (ALT) sheep are indigenous breeds adapted to extreme heat and severe cold in their respective native environments. However, the mechanisms underlying their divergent meat quality remain unclear. Using *longissimus dorsi* muscle from 15 TBL and 15 ALT sheep, we integrated phenotypic evaluation with non-targeted metabolomics and proteomics to elucidate the impact of environmental adaptation on ovine meat quality. Compared to the cold-adapted ALT sheep, the heat-tolerant TBL sheep exhibited lower post-mortem pH, reduced cooking loss, smaller muscle fiber cross-sectional area, and elevated selenium and magnesium levels. Multi-omics identified 99 differentially expressed proteins and 364 differentially expressed metabolites. Core divergence was enriched in lipid and amino acid metabolism and stress response networks, particularly the Apelin signaling, glycerophospholipid metabolism, and ferroptosis pathways. Lipid remodeling driven by glycerophospholipid metabolism emerged as a critical bridge linking adaptation to meat quality. Notably, glycero-3-phosphocholine, regulated by GPCPD1 and related enzymes, maintained cell membrane homeostasis and osmotic pressure, thereby enhancing water-holding capacity and tenderness. These findings reveal the multi-omics basis of climate-driven divergence in ovine meat quality, offering theoretical support for breeding stress-resilient, high-quality indigenous sheep breeds in extreme environments.

## 1. Introduction

As a major source of high-quality protein in the human diet, lamb meat quality traits directly determine both eating quality and economic value. Meat quality is a complex, multifactorial trait comprising key indicators such as meat color, tenderness, cooking loss, intramuscular fat content, and muscle fiber characteristics. The development of these attributes is regulated by the interplay of genetics, nutrition, husbandry management, and environmental factors [1,2,3]. Among environmental stressors, extreme temperature variations, including both severe heat and cold stress, represent primary constraints on animal health and productive performance. Under chronic thermal or cold stress, adaptive physiological mechanisms lead to metabolic shifts that subsequently alter lipid deposition capacity and ultimately affect meat quality attributes [4,5]. The mechanisms by which environmental factors influence livestock production performance and meat quality development have garnered increasing research attention. For instance, *Bos indicus* cattle can adapt to heat stress through metabolic adjustments such as reduced protein degradation rates and optimized mitochondrial function. However, the concomitant upregulation of calpastatin activity limits post-mortem proteolysis, thereby reducing beef tenderness [6]. Specific forages have also been shown to modulate the fatty acid composition of lamb muscle, thereby influencing flavor and oxidative stability. The distinctive quality of geographically protected authentic lamb is intimately linked to local environmental factors, including pasture composition and climate, underscoring that breed-specific foraging behavior and metabolic adaptation collectively determine divergent meat quality outcomes [7]. Therefore, elucidating the regulatory role of extreme climatic adaptation mechanisms on muscle physiology is critical for deciphering inter-breed differences and enhancing the productive performance of indigenous livestock.

China harbors abundant indigenous sheep germplasm resources, many of which have evolved unique adaptations to extreme environments through prolonged natural and artificial selection. Turpan Black sheep is an indigenous meat-purpose breed developed in the Turpan region of Xinjiang, with a stable genetic lineage derived from the Mongolian Bayanbulak fat-tailed sheep, Kazakh sheep, and Karakul sheep [8]. This breed exhibits remarkable tolerance to ambient temperatures exceeding 42 °C in summer [9]. It produces high-quality lamb characterized by elevated protein content, low fat and cholesterol levels, and abundant mineral concentrations under coarse-feeding conditions [10,11]. Previous studies have demonstrated that TBL sheep maintain superior reproductive performance under high-temperature conditions, with physiological indices significantly exceeding those of Kazakh sheep subjected to concurrent heat stress [8]. While heat stress severely impacts livestock, extreme cold stress similarly challenges animal survival and muscle metabolism. The ALT, an indigenous fat-tailed breed from northern Xinjiang, has evolved robust physiological mechanisms to endure severe winter cold (temperatures plummeting to −40 °C) and forage scarcity. As a dual-purpose coarse-wool breed, ALT sheep are distinguished by their well-developed fat tail and rump, enabling substantial fat accumulation during the withered-grass season, which contributes to their tender and distinctive meat flavor [12]. Using iTRAQ-based proteomics, Wang et al. [13] analyzed fat deposition in the adipose tissue of ALT sheep, delineating the molecular regulatory networks governing lipid storage and mobilization. Comparing the heat-tolerant TBL sheep with the cold-adapted ALT sheep provides an ideal natural model. Rather than utilizing a highly controlled commercial line, investigating these two distinct indigenous breeds within their respective native environments allows us to specifically explore how opposite extremes of natural climatic adaptation shape biological divergence in muscle metabolism and ultimate meat quality.

How skeletal muscle in these indigenous breeds responds physiologically to their respective extreme environments and, consequently, influences meat quality development warrants in-depth investigation. In recent years, high-throughput omics technologies, particularly proteomics and metabolomics, have been widely applied to elucidate the molecular underpinnings of meat quality [14]. Proteins are the direct executors of physiological functions, whereas metabolites accurately reflect the terminal phenotypic state. The combined analysis of these two layers facilitates a systematic elucidation of biological processes at the expression and metabolic regulatory network levels, offering an effective approach to dissect the impact of environmental adaptation on muscle physiology [15]. The *longissimus dorsi* muscle exhibits a uniform fiber-type distribution, stable meat quality characteristics, and sensitive metabolic responses to environmental stressors, rendering it a suitable tissue for evaluating muscle physiological divergence under contrasting adaptive regimes [16]. In the present study, we selected the extreme heat-adapted TBL sheep and the severe cold-adapted ALT sheep as experimental models. Through combined proteomic and metabolomic analyses, we systematically compared the molecular profiles, meat quality traits, and histological characteristics of the *longissimus dorsi* muscle. The primary objective was to elucidate the multi-omics mechanisms underlying meat quality divergence driven by contrasting climatic adaptations, thereby providing a theoretical foundation for research on environmental adaptation mechanisms and the utilization of indigenous livestock germplasm resources.

## 2. Materials and Methods

### 2.1. Experimental Animals and Sample Collection

All animal experimental procedures in this study were conducted in strict compliance with the regulations of the Experimental Animal Ethics Committee of Xinjiang Agricultural University (Approval No.: 2023053). Fifteen 12-month-old Turpan Black sheep and fifteen 12-month-old Altay sheep were obtained from the Toksun County State-Owned Pasture in Turpan Prefecture and the Fuhai County Breeding Sheep Farm in Altay Prefecture, respectively. Both breeds were raised under their respective traditional natural grazing systems on state-owned pastures without additional concentrate supplementation, relying entirely on natural native forage. Specifically, the Toksun pasture is characterized by a low altitude and an extremely arid climate, with summer temperatures frequently exceeding 40 °C. In contrast, the Fuhai farm is situated at a higher altitude and experiences severe winter cold, with temperatures often dropping below −20 °C. All individuals were used for subsequent meat quality assessment, proteomic analysis, and metabolomic profiling. Prior to slaughter, all sheep were fasted for 12 h with free access to water for the final 2 h, and pre-slaughter live weight was recorded. Carcass weight was measured immediately post-slaughter. Within 45 min after exsanguination, the pH of the *longissimus dorsi* muscle was determined using a portable pH meter. Simultaneously, *longissimus dorsi* muscle samples were collected from the same side between the 12th and 13th ribs of each sheep and designated as the TBL-LD group and the ALT-LD group (representing the thoracic portion of the longissimus muscle). Samples for proteomic and metabolomic analyses were immediately snap-frozen in liquid nitrogen within 45 min post-mortem and subsequently stored at −80 °C. For histological analysis, adjacent muscle samples collected at 45 min post-mortem were directly fixed in 4% paraformaldehyde. To assess conventional meat quality traits, the remaining intact *longissimus dorsi* muscle was chilled at 4 °C for 24 h.

### 2.2. Meat Quality Measurements and Histological Analysis

Conventional meat quality traits (color, cooking loss, and shear force) were evaluated at 24 h post-mortem after chilling at 4 °C, in accordance with the relevant national food safety standards of China. Post-mortem pH was measured at three distinct sites per sample using a portable pH meter equipped with a penetration glass electrode (Testo 205, Testo AG, Lenzkirch, Germany), calibrated with standard buffer solutions at pH 4.0 and 7.0, and the mean value was calculated. Meat color parameters in the CIELAB color space (L*, a*, b*) were determined on the freshly cut cross-sectional surface of the muscle using a portable colorimeter (CR-400, Konica Minolta, Tokyo, Japan). Cooking loss was evaluated via the direct water bath method: samples were weighed (W_1_), heated in an 85°C water bath for 30 min, cooled to ambient temperature, blotted dry of surface moisture, and reweighed (W_2_). Cooking loss (%) was calculated using the formula: (W_1_ − W_2_)/W_1_ × 100. Meat tenderness was objectively evaluated by measuring the Warner-Bratzler Shear Force. Specifically, Shear force was measured using a texture analyzer (TA.XT Plus, Stable Micro Systems, Surrey, UK) equipped with a Warner–Bratzler shear blade on 1 cm × 1 cm × 1 cm meat cores excised parallel to the muscle fiber orientation. Measurements were performed at a test speed of 1.0 mm/s with three replicates per sample, and the mean maximum force was expressed in Newtons (N). Proximate composition (crude fat and crude protein) and mineral element concentrations (iron, magnesium, zinc, copper, selenium, calcium) were determined using conventional analytical methods.

Skeletal muscle histomorphology was examined using hematoxylin and eosin (HE) staining. Paraffin-embedded sections were prepared from *longissimus dorsi* samples that had been fixed in 4% paraformaldehyde. Muscle fiber architecture was preliminarily visualized under a light microscope, and ImageJ software (version 1.53t, National Institutes of Health, Bethesda, MD, USA) was employed for quantitative analysis of muscle fiber diameter and cross-sectional area (CSA).

### 2.3. Proteomic Analysis

#### 2.3.1. Protein Extraction

Proteins were extracted using a lysis buffer method. Briefly, frozen samples were retrieved from −80 °C storage and ground into a fine powder using liquid nitrogen. A 50 mg aliquot of the powdered tissue was transferred to a 1.5 mL centrifuge tube, to which lysis buffer (containing 8 M urea, 1 mM PMSF, and 2 mM EDTA) was added. The mixture was subjected to ultrasonication on ice for 5 min, followed by centrifugation at 15,000× *g* for 10 min at 4 °C. The resulting supernatant was collected, and protein concentration was determined using a BCA Protein Assay Kit (Beyotime Biotechnology, Shanghai, China).

#### 2.3.2. Protein Digestion

A 100 μg aliquot of the protein solution (adjusted according to the measured concentration) was diluted to a final volume of 200 μL with 8 M urea. The sample was reduced with dithiothreitol (DTT) at a final concentration of 5 mM for 45 min at 37 °C, followed by alkylation with iodoacetamide (IAA) at a final concentration of 11 mM for 15 min at room temperature in the dark. Subsequently, 800 μL of 25 mM ammonium bicarbonate solution and 2 μL of trypsin (V5280, Promega, Madison, WI, USA) were added, and the mixture was incubated overnight at 37 °C for digestion. The resulting peptide solution was acidified to pH 2–3 with 20% trifluoroacetic acid (TFA) and desalted using C18 resin (Millipore, Billerica, MA, USA). Peptide concentration was determined using a Pierce™ Quantitative Peptide Assay Kit (including standards; Thermo Fisher Scientific, Waltham, MA, USA).

#### 2.3.3. Nano-LC-MS/MS Analysis

Chromatographic separation for data-independent acquisition (DIA) analysis was performed using a Vanquish Neo UHPLC system (Thermo Fisher Scientific). Mobile phase A consisted of 0.1% formic acid in water, and mobile phase B consisted of 0.1% formic acid in acetonitrile (100% acetonitrile). Peptides were trapped on a PepMap Neo Trap Cartridge (300 μm × 5 mm, 5 μm particle size) and separated on an Easy-Spray™ PepMap™ Neo UHPLC analytical column (150 μm × 15 cm, 2 μm particle size) using a trap-and-elute configuration. The analytical column temperature was maintained at 55 °C via an integrated column oven. The injection amount was 200 ng, and the flow rate was set to 2.5 μL/min. The effective gradient duration was 6.9 min, with a total run time of 8 min.

The nano-UHPLC eluate was directly interfaced with an Orbitrap Astral high-resolution mass spectrometer (Thermo Fisher Scientific, Waltham, MA, USA) operated in positive-ion mode for DIA mass spectrometric analysis. Survey full-scan MS spectra were acquired over an *m*/*z* range of 380–980 at a resolution of 240,000 (at *m*/*z* 200), with a normalized AGC target of 500% and a maximum injection time of 5 ms. MS/MS acquisition was performed in DIA mode with 299 isolation windows, each with a 2 Th isolation width. Higher-energy collisional dissociation (HCD) was applied at a collision energy of 25%, with a normalized AGC target of 500% and a maximum injection time of 3 ms for MS/MS scans.

#### 2.3.4. Database Searching and Quantification

Raw DIA mass spectrometric data were processed using DIA-NN software (version 1.8.1) in library-free mode. The search parameters were as follows: the sequence database employed was the UniProtKB *Ovis aries* (sheep) proteome (UP000002356, downloaded on 25 September 2024, containing 23,108 entries). Deep-learning-based spectral library prediction was enabled. Match-between-runs (MBR) was activated to generate a spectral library from the DIA data, which was subsequently used for re-analysis of the DIA data to obtain both qualitative and quantitative protein-level results. Precursor and protein-level identifications were filtered at a false discovery rate (FDR) of 1%. The filtered dataset was retained for subsequent bioinformatic analyses.

### 2.4. Non-Targeted Metabolomic Analysis

#### 2.4.1. Metabolite Extraction

Frozen samples stored at −80 °C were thawed on ice (n = 15 per breed) and ground into a fine powder using liquid nitrogen. A 20 mg aliquot of the ground tissue was transferred to a tube, to which 400 μL of an extraction solvent (methanol/water = 7:3, v/v) containing an internal standard was added. The mixture was vortexed at 1500 rpm for 5 min, incubated on ice for 15 min, and subsequently centrifuged at 12,000 rpm for 10 min at 4 °C. A 300 μL volume of the resulting supernatant was collected and stored at −20 °C for 30 min. The sample was then centrifuged again at 12,000 rpm for 3 min at 4 °C, after which a 200 μL aliquot of the final supernatant was transferred for LC-MS analysis.

#### 2.4.2. UHPLC-MS/MS Analysis

LC-MS/MS analysis was performed using a UHPLC system (Vanquish, Thermo Fisher Scientific) coupled with a Q Exactive HF-X hybrid quadrupole-Orbitrap high-resolution mass spectrometer (Orbitrap MS, Thermo Fisher Scientific). Chromatographic separation was achieved on a Waters ACQUITY Premier HSS T3 column (1.8 μm, 2.1 mm × 100 mm). The mobile phase consisted of 0.1% formic acid in water (solvent A) and 0.1% formic acid in acetonitrile (solvent B). The gradient elution program was as follows: 5% to 20% B over 2 min, increased to 60% B over the next 3 min, further increased to 99% B over 1 min and held for 1.5 min, then returned to 5% B over 0.1 min and maintained for 2.4 min. The column temperature was maintained at 40 °C, the flow rate was set to 0.4 mL/min, and the injection volume was 4 μL. An additional aliquot was analyzed in negative-ion mode using the same elution gradient. All analyses were performed in full-scan MS and data-dependent MS/MS (dd-MS^2^) acquisition mode with dynamic exclusion. Mass spectrometric detection employed an electrospray ionization (ESI) source operated in both positive and negative ion modes. Full-scan spectra were acquired over an *m*/*z* range of 75–1000 at a resolution of 35,000. Additional MS parameters were as follows: ion spray voltage, 3.5 kV (positive mode) or 3.2 kV (negative mode); sheath gas flow rate, 30 arbitrary units (Arb); auxiliary gas flow rate, 5 Arb; ion transfer tube temperature, 320 °C; vaporizer temperature, 300 °C; collision energy, 30, 40, and 50 V (stepped); signal intensity threshold, 1 × 10^6^ counts per second (cps); Top N vs. Top speed setting, 10; and dynamic exclusion duration, 3 s.

#### 2.4.3. Data Processing

Raw mass spectrometric data files were converted to mzML format using ProteoWizard. Peak detection, alignment, and retention time correction were performed using the XCMS program. To ensure data quality while preserving biologically relevant presence/absence variation, peak filtering was applied per group. Features with a missing rate exceeding 50% in all sample groups were filtered out. For the retained features, missing values were imputed using a hybrid strategy: the k-nearest neighbors (KNN) algorithm was utilized for features with <50% missingness within a group, whereas 1/5 of the minimum positive value of the variable was assigned for imputation when the missingness exceeded 50% (indicating values likely below the limit of detection). Peak areas were subsequently normalized using support vector regression (SVR). Metabolite identification was conducted by searching the filtered and corrected peaks against an in-house database, integrated public spectral libraries, predictive libraries, and the metDNA annotation method. Features with a composite identification score ≥0.5 and a coefficient of variation (CV) <0.3 in quality control (QC) samples were retained. Finally, data from positive and negative ionization modes were merged by prioritizing the identification with the highest qualitative confidence level and the lowest CV, yielding the final dataset.

### 2.5. Differential Analysis

To identify significantly altered protein expression, differentially expressed proteins (DEPs) were defined using a threshold of fold change (FC) >1.5 or <0.67 and *p* < 0.05 as determined by Student’s *t*-test. For metabolomic data, differential metabolites (DEMs) between the two groups were determined based on variable importance in projection (VIP) scores (VIP > 1) derived from orthogonal partial least squares-discriminant analysis (OPLS-DA) and *p*-value < 0.05 from Student’s *t*-test. Although multiple-comparison correction using the Benjamini–Hochberg FDR method was performed to monitor the global error rate, the nominal *p*-value was retained for final feature selection to minimize Type II errors (false negatives) in this exploratory, discovery-driven multi-omics study. To strictly control for false positives, these nominal *p*-value thresholds were combined with stringent orthogonal filters (FC and VIP scores) and subsequent downstream biological pathway cross-validation. Prior to OPLS-DA, data were log_2_-transformed and mean-centered. Score plots and permutation plots were generated using the R package MetaboAnalystR. Permutation testing (200 permutations) was conducted to validate the model and prevent overfitting.

### 2.6. Bioinformatic Analysis

To gain comprehensive insight into the functional classification and biological characteristics of the differentially expressed proteins (DEPs), Gene Ontology (GO) enrichment analysis was performed on the identified protein sequences using the ClusterProfiler package, with a significance threshold of *p* ≤ 0.05. DEPs and differentially expressed metabolites (DEMs) were annotated using the Kyoto Encyclopedia of Genes and Genomes (KEGG) Compound database (http://www.kegg.jp/kegg/compound/) (accessed on 28 February 2025), and annotated metabolites were subsequently mapped to the KEGG Pathway database (http://www.kegg.jp/kegg/pathway.html) (accessed on 28 February 2025). Direct and indirect interactions among target proteins were identified using the STRING database (http://string-db.org/) (accessed on 28 February 2025), and the resulting interaction networks were constructed and visualized in Cytoscape (version 3.9.1).

### 2.7. Statistical Analysis

Data were compiled using Microsoft Excel. All statistical comparisons between the two groups were performed using independent-samples Student’s *t*-tests in IBM SPSS Statistics version 27.0 (Chicago, IL, USA). Quantitative data are expressed as mean ± standard deviation (SD). Statistical significance was assessed at *p* < 0.05 and *p* < 0.01.

## 3. Results

### 3.1. Differences in Meat Quality Traits and Histomorphology of the Longissimus Dorsi Muscle Between Turpan Black and Altay Sheep

**Figure 1:**
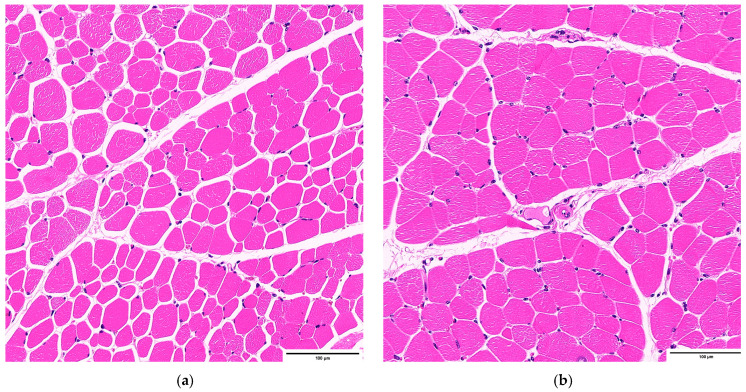

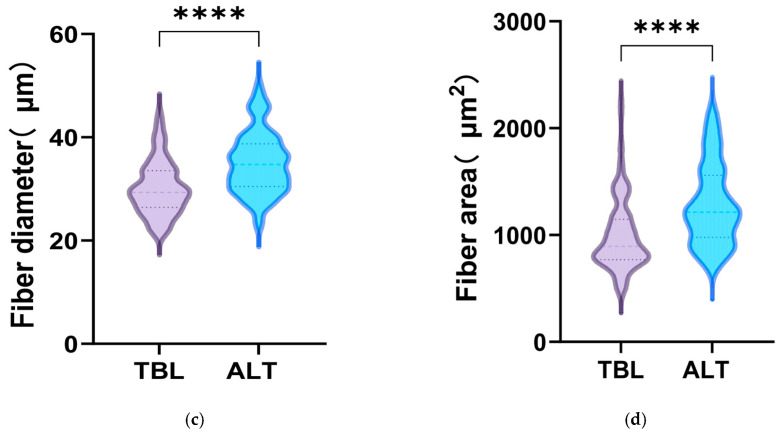
Histomorphological characteristics of the *longissimus dorsi* muscle in TBL and ALT sheep. (**a**) Representative histological section of *longissimus dorsi* muscle from TBL sheep; (**b**) Representative histological section of *longissimus dorsi* muscle from ALT sheep; (**c**) Violin plot of muscle fiber diameter in the *longissimus dorsi* of TBL and ALT sheep; (**d**) Violin plot of muscle fiber CSA in the *longissimus dorsi* of TBL and ALT sheep. Statistical significance is denoted as **** *p* < 0.0001. The three black dashed lines ascending from the bottom of each violin plot represent the lower quartile (25th percentile), the median (50th percentile), and the upper quartile (75th percentile), respectively.

To systematically compare meat quality differences between TBL and ALT sheep, we evaluated conventional meat quality parameters, mineral element concentrations, and muscle fiber histological characteristics of the *longissimus dorsi* muscle. Among conventional meat quality traits (Table 1), the pH value and cooking loss percentage of TBL sheep were significantly lower than those of ALT sheep (*p* < 0.01), indicating superior water-holding capacity in the TBL breed; no significant differences were observed for the remaining parameters. Regarding mineral composition (Table 2), TBL muscle exhibited significantly higher concentrations of selenium (*p* < 0.01) and magnesium (*p* < 0.05), whereas iron (*p* < 0.01) and aluminum (*p* < 0.05) contents were significantly lower compared with ALT sheep, suggesting potential advantages in nutritional value and food safety. Histological analysis (Figure 1a–d) further revealed that both muscle fiber diameter and CSA were significantly smaller in TBL sheep than in ALT sheep (*p* < 0.01), indicative of a finer muscle structure. Collectively, these findings demonstrate that TBL and ALT sheep exhibit divergent meat quality attributes, with TBL primarily characterized by a finer muscle fiber structure, enhanced water-holding capacity, and marked enrichment of the trace element Se and the macromineral Mg.

### 3.2. Proteomic Characteristics of Turpan Black and Altay Sheep

#### 3.2.1. Protein Identification and Quantification

**Figure 2:**
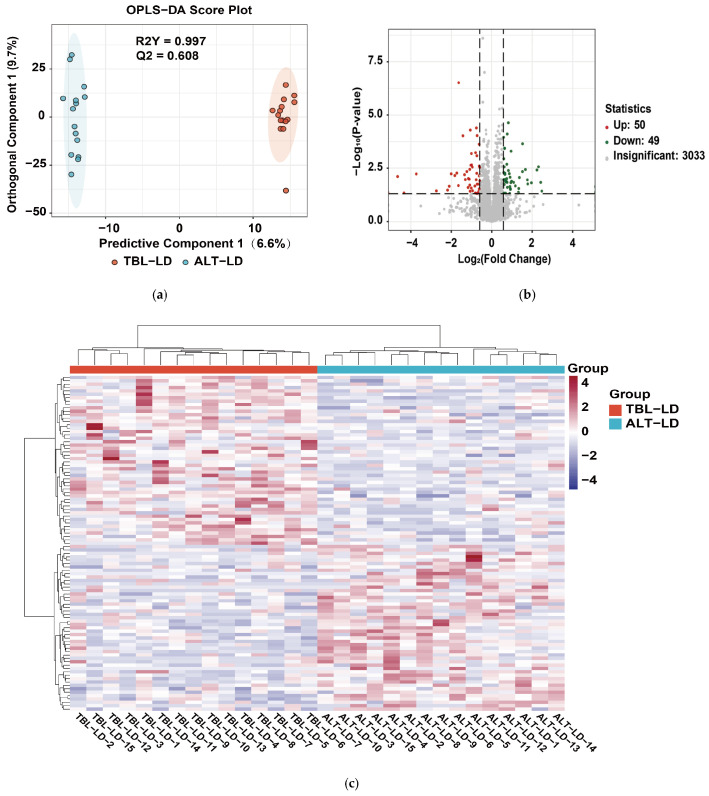
Analysis of differentially expressed proteins in the *longissimus dorsi* muscle of TBL and ALT sheep. (**a**) OPLS-DA score plot of proteomic profiles from the *longissimus dorsi* muscle of TBL and ALT sheep. the orange dots represent the TBL sheep, and the light blue dots represent the ALT sheep; (**b**) Volcano plot of DEPs between TBL and ALT sheep. (**c**) Heatmap of DEPs in the *longissimus dorsi* muscle of TBL and ALT sheep. Red dots or regions indicate upregulated DEPs, blue dots or regions indicate downregulated DEPs, and gray dots represent proteins with no significant differential expression.

To characterize the skeletal muscle proteome profiles of Turpan Black and Altay sheep, we performed high-throughput qualitative and quantitative analysis of *longissimus dorsi* muscle protein extracts using ultra-high-performance liquid chromatography coupled with data-independent acquisition quadrupole-Orbitrap mass spectrometry (UHPLC-DIA-QE-MS). Based on the quantitative data, a supervised OPLS-DA model was initially constructed. As illustrated in Figure 2a, the two groups exhibited distinct and well-resolved clustering in the principal component projection space without any overlap. The model yielded an explanatory power (R^2^Y) of 0.997 and a predictive capability (Q^2^) of 0.608. The OPLS-DA model was rigorously validated using a 200-permutation test, which yielded significant results (empirical *p*-value < 0.05 for both R^2^Y and Q^2^), confirming that the high R^2^ value was not due to overfitting. This indicates excellent goodness-of-fit and robustness, confirming substantial proteome-level divergence in skeletal muscle between the two breeds and providing a reliable foundation for subsequent differential expression analysis.

Based on the above proteomic divergence, 3132 quantifiable proteins were subjected to differential expression analysis. Using a significance threshold of *p* < 0.05 and a fold change (FC) ≥1.5 (upregulated) or ≤0.67 (downregulated), 99 DEPs were identified, comprising 50 upregulated and 49 downregulated proteins. The volcano plot (Figure 2b) graphically depicts the distribution of these DEPs in terms of both statistical significance and magnitude of change, highlighting the overall pattern of protein abundance alterations between the two breeds. To more clearly delineate the expression profiles of the DEPs, Z-score normalization was applied to their quantitative values, and hierarchical clustering analysis was performed, with results displayed as a heatmap (Figure 2c). The heatmap revealed highly consistent expression clustering among biological replicates within each breed, whereas pronounced inverse trends were evident between TBL and ALT sheep. Several proteins exhibited breed-specific patterns of elevated or diminished expression. These findings not only corroborate the significance and reproducibility of intergroup differences at the global expression level but also suggest that these DEPs represent core molecular targets potentially mediating the divergence in muscle physiological traits and meat quality phenotypes between the two breeds, thereby providing a critical foundation for subsequent in-depth mechanistic elucidation.

#### 3.2.2. GO Functional and KEGG Pathway Enrichment Analysis of DEPs

**Figure 3:**
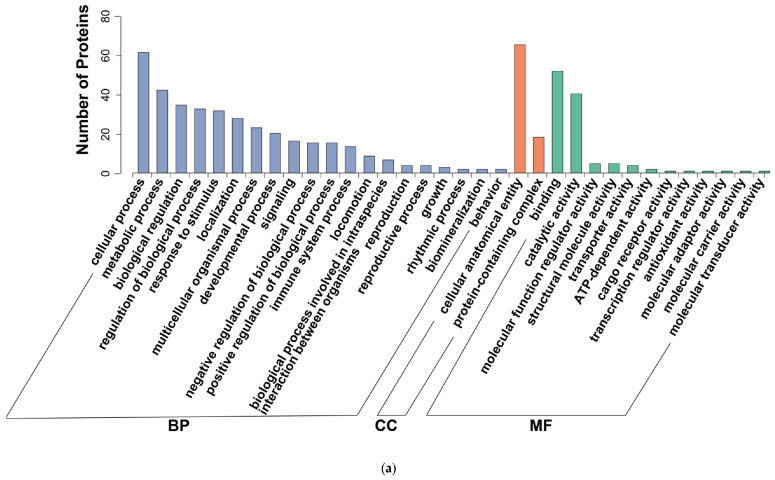

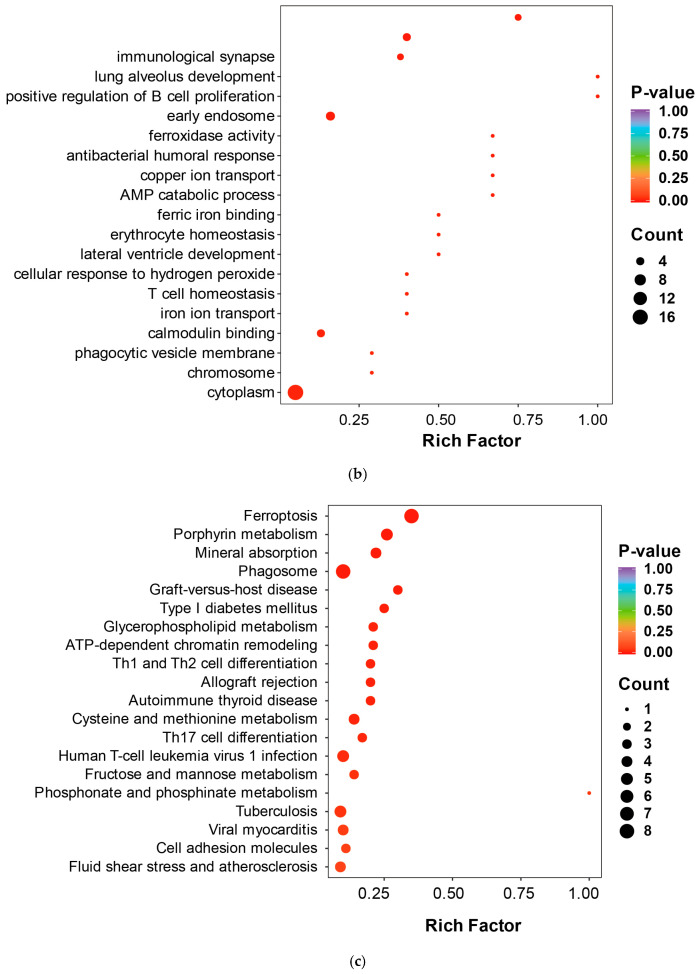
GO functional and KEGG pathway enrichment analysis of DEPs between TBL and ALT sheep. (**a**) Bar chart of GO annotations for DEPs; (**b**) Bubble plot of GO enrichment analysis for DEPs. (**c**) Bubble plot of KEGG pathway enrichment analysis for DEPs.

To elucidate the molecular mechanisms underlying the DEPs associated with meat quality differences between TBL and ALT sheep, GO enrichment analysis was performed (Figure 3). A total of 625 GO terms across the three major categories, biological process (BP), cellular component (CC), and molecular function (MF), were significantly enriched (Figure 3a). The top 20 significantly enriched GO terms (ranked by count number, *p* < 0.05) were visualized in a bubble plot (Figure 3b). Within the BP category, cellular iron ion homeostasis, AMP catabolic process, and iron ion transport were among the core enriched terms. The CC category was predominantly enriched in immunological synapse, early endosome, and cytoplasm, whereas the MF category showed significant enrichment in potassium channel activity, ferroxidase activity, and ferric iron binding. These findings indicate that the DEPs between the two breeds are centrally involved in iron homeostasis and transport, energy metabolism, ion channel regulation, and redox-associated processes.

To further investigate the molecular basis of divergent meat quality and metabolic physiology between the TBL-LD and ALT-LD groups, KEGG pathway enrichment analysis was conducted on the identified DEPs. The results revealed significant enrichment in multiple pathways closely associated with oxidative stress and meat quality regulation (Figure 3c). Specifically, pathways related to redox homeostasis and cell death, including ferroptosis, porphyrin metabolism, mineral absorption, and cysteine and methionine metabolism, were significantly enriched. Additionally, pathways involved in membrane structure and energy metabolism, such as glycerophospholipid metabolism and fructose and mannose metabolism, also exhibited significant enrichment. Collectively, the KEGG pathway enrichment analysis demonstrates that the core molecular divergence between TBL-LD and ALT-LD is primarily manifested in three aspects: antioxidant defense mechanisms, trace element utilization, and key flavor precursor metabolism. The differential regulation of these pathways underpins the distinct meat quality and metabolic physiological traits that characterize the adaptation of these two sheep breeds to contrasting climatic environments.

#### 3.2.3. Protein–Protein Interaction (PPI) Analysis

**Figure 4:**
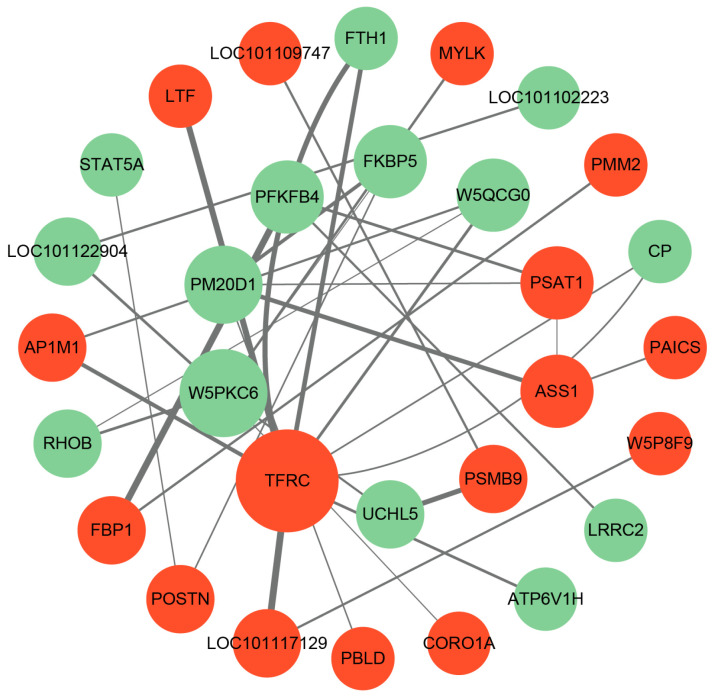
PPI network analysis of DEPs in the *longissimus dorsi* muscle of TBL and ALT sheep. Node size is positively correlated with degree value. Edge thickness represents the combined interaction score, with thicker edges indicating higher confidence of interaction. Red nodes denote proteins upregulated in TBL sheep, and green nodes denote proteins downregulated in TBL sheep (upregulated in ALT sheep).

To investigate the functional associations among DEPs identified in the *longissimus dorsi* muscle of TBL and ALT sheep, a PPI network was constructed using the STRING database (https://cn.string-db.org/) and visualized with Cytoscape software. As shown in Figure 4, the resulting PPI network comprised 34 nodes and 58 edges, corresponding to 34 proteins and 58 pairwise interactions. Network topology analysis revealed that six DEPs, transferrin receptor (TFRC), lactotransferrin (LTF), phosphoserine aminotransferase 1 (PSAT1), fructose-1,6-bisphosphatase 1 (FBP1), ceruloplasmin (CP), and N-fatty-acyl-amino acid synthase/hydrolase (PM20D1) occupied highly connected hub positions, each exhibiting strong interactions with three to five other proteins. This observation suggests that these hub proteins may play pivotal regulatory roles in the formation of breed-specific meat quality traits. Notably, a robust interaction subnetwork comprising TFRC, AP1M1, LTF, CP, FTH1, ATP6V1H, and LOC101117129 constitutes a core regulatory module for iron metabolism, wherein TFRC and LTF cooperatively govern iron homeostasis.

#### 3.2.4. Correlation Analysis Between Meat Quality Traits and DEPs

**Figure 5:**
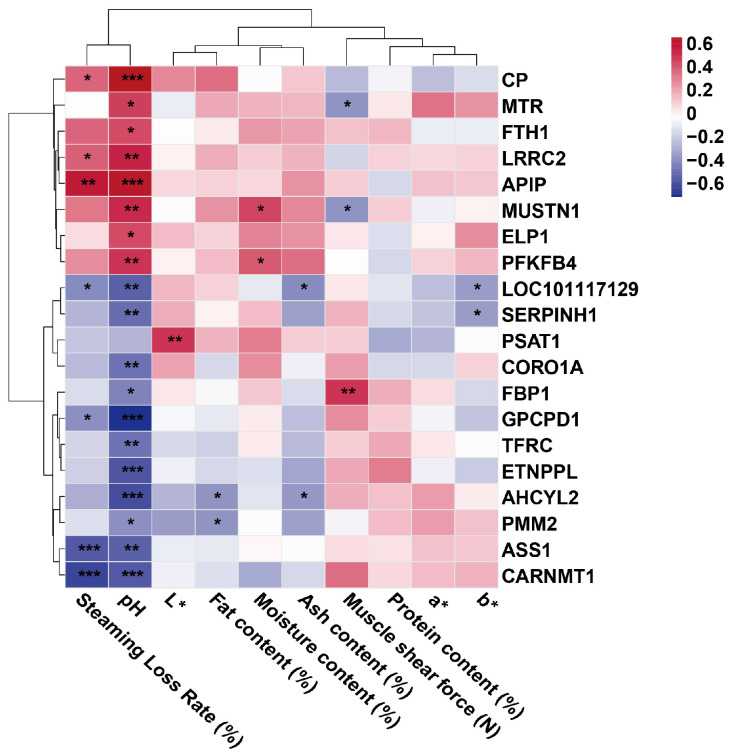
Correlation heatmap between meat quality traits and DEPs. The color intensity of each tile represents the magnitude of the Pearson correlation coefficient (r): red denotes positive correlation, and blue denotes negative correlation. Statistically significant correlations are denoted as * *p* < 0.05, ** *p* < 0.01, and *** *p* < 0.001.

To further identify key DEPs closely associated with meat quality traits, Pearson correlation analysis was performed between the relative quantification values of 20 core DEPs and various meat quality parameters. This analysis aimed to delineate the extent of association between protein expression and meat quality phenotypes, thereby providing a foundation for elucidating the potential mechanisms by which DEPs regulate meat quality traits. The results revealed substantial variation in both the number of associated DEPs and the nature of their correlations across different meat quality parameters (Figure 5). Among the 20 DEPs analyzed, three exhibited significant correlations with meat color parameters (L, b*); three were significantly correlated with shear force; seven were significantly correlated with cooking loss; nineteen were significantly correlated with pH value; additionally, two DEPs each showed significant correlations with fat, moisture, and ash content.

Specifically, with respect to water-holding capacity, cooking loss exhibited a highly significant positive correlation with APIP (r = 0.56, *p* < 0.01) and significant positive correlations with CP and LRRC2 (r = 0.36 and 0.37, respectively; *p* < 0.05). Conversely, cooking loss showed highly significant negative correlations with CARNMT1 and ASS1 (r = −0.65 and −0.58, respectively; *p* < 0.001), as well as significant negative correlations with LOC101117129 and GPCPD1 (r = −0.43 and −0.41, respectively; *p* < 0.05). Regarding pH value, significant positive correlations were observed with APIP and CP (r = 0.60 and 0.64, respectively; *p* < 0.001), and highly significant positive correlations were found with LRRC2, MUSTN1, and PFKFB4 (r = 0.52, 0.51, and 0.48, respectively; *p* < 0.01). In contrast, GPCPD1, AHCYL2, and ETNPPL displayed highly significant negative correlations with pH value (r = −0.73, −0.62, and −0.59, respectively; *p* < 0.001), while ASS1, SERPINH1, and TFRC also exhibited highly significant negative correlations with pH (r = −0.56, −0.52, and −0.50, respectively; *p* < 0.01).

Notably, among all key DEPs examined, each protein displayed statistically significant correlations with at least one meat quality trait, underscoring the critical regulatory roles these proteins play in meat quality development. The observation that pH-associated DEPs constituted the largest proportion suggests that these key differential proteins may systematically participate in and modulate the ultimate formation of core meat quality attributes—such as meat color and water-holding capacity primarily through influencing post-mortem pH homeostasis.

### 3.3. Metabolomic Differences Between Turpan Black and Altay Sheep

#### 3.3.1. Metabolite Identification

**Figure 6:**
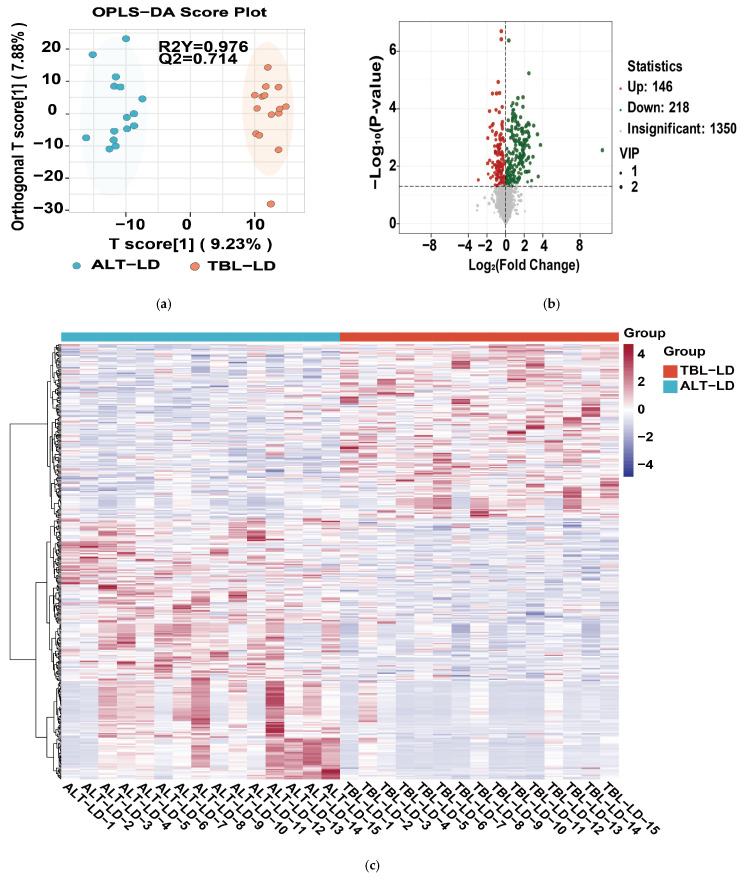
Analysis of differentially expressed metabolites in the *longissimus dorsi* muscle of TBL and ALT sheep (**a**) OPLS-DA score plot of metabolite profiles from the *longissimus dorsi* muscle of TBL and ALT sheep. (**b**) Volcano plot displaying DEMs between TBL and ALT sheep. (**c**) Heatmap of differentially expressed metabolites in the *longissimus dorsi* muscle of TBL and ALT sheep.

To investigate breed-specific differences in the skeletal muscle metabolic profiles of TBL and ALT sheep and their potential biological implications, we performed non-targeted metabolomic analysis of *longissimus dorsi* muscle samples using liquid chromatography–tandem mass spectrometry (LC–MS/MS). A total of 1714 metabolites were identified across all samples. To globally delineate the metabolic divergence between the two groups, the OPLS-DA model was constructed. As shown in Figure 6a, the two groups exhibited clear and well-resolved clustering in the principal component projection space without any overlap. The model yielded an R^2^Y of 0.976 and a Q^2^ of 0.714. The OPLS-DA model was rigorously validated using a 200-permutation test, which yielded significant results (empirical *p*-value < 0.05 for both R^2^Y and Q^2^), confirming that the high R^2^ value was not due to overfitting. These metrics exceed the conventional thresholds for model robustness, thereby confirming excellent goodness-of-fit and predictive performance and providing a reliable statistical foundation for subsequent differential metabolite screening.

Based on the validated model, a combined univariate and multivariate statistical approach was employed for in-depth identification of differential metabolites. Using VIP > 1 and *p* < 0.05 as significance criteria, a total of 364 DEMs were identified. The volcano plot (Figure 6b) visually depicts the overall distribution of these DEMs in terms of both statistical significance and fold-change magnitude. Compared with ALT sheep, 146 metabolites were significantly upregulated, and 218 metabolites were significantly downregulated in the *longissimus dorsi* muscle of TBL sheep. Subsequently, the peak areas of the 364 DEMs were Z-score normalized and subjected to hierarchical clustering analysis, with the results displayed as a heatmap (Figure 6c). The heatmap revealed highly consistent metabolic expression patterns among biological replicates within each breed, whereas pronounced inverse trends were evident between the two breeds, with several metabolites exhibiting distinct breed-specific accumulation or depletion profiles. Collectively, these findings demonstrate substantial differences in the skeletal muscle metabolome between the two sheep breeds, thereby offering critical metabolomic insights and candidate targets for elucidating the molecular mechanisms underlying breed-specific divergence in meat quality traits.

#### 3.3.2. KEGG Pathway Enrichment Analysis of Differential Metabolites Between TBL and ALT Sheep

**Figure 7:**
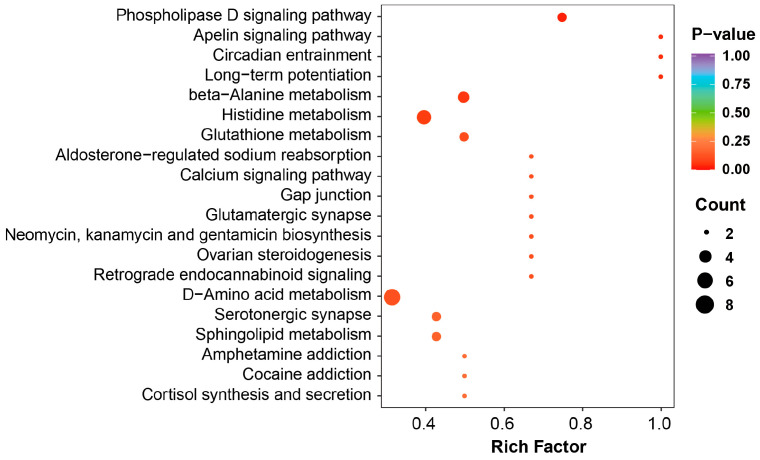
Bubble plot of KEGG pathway enrichment analysis for differentially expressed metabolites between TBL and ALT sheep.

To elucidate the potential physiological functions of DEMs underlying breed-specific differences between TBL and ALT sheep, KEGG pathway enrichment analysis was performed. The results revealed that, compared with the ALT breed, the TBL breed exhibited significant enrichment (*p* < 0.05) and upregulation in four distinct pathways: the Apelin signaling pathway, phospholipase D signaling pathway, long-term potentiation, and circadian entrainment (Figure 7). Key metabolites enriched within these pathways included sphingosine-1-phosphate, L-glutamate, and cyclic adenosine monophosphate (cAMP). The Apelin and phospholipase D signaling pathways are broadly implicated in the regulation of energy metabolism, cell proliferation, and stress responses, whereas long-term potentiation and circadian entrainment are associated with neuronal signal transduction and physiological rhythm regulation. The activation states of these pathways collectively suggest that TBL sheep may possess distinctive characteristics in cellular energy metabolism, signal transduction, and the coordination of overall physiological rhythms, thereby indirectly influencing the development of meat quality traits.

#### 3.3.3. Correlation Analysis Between Meat Quality Traits and DEMs

**Figure 8:**
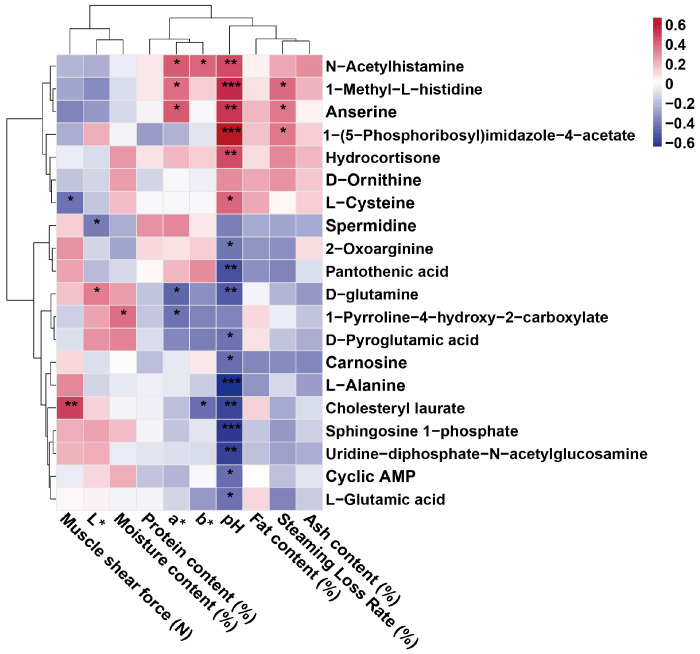
Correlation heatmap between meat quality traits and DEMs. Color intensity of each tile represents the magnitude of the Pearson correlation coefficient (r): red denotes positive correlation, and blue denotes negative correlation. Statistically significant correlations are denoted as * *p* < 0.05, ** *p* < 0.01, and *** *p* < 0.001.

To investigate the specific impact of metabolic alterations on meat quality traits, Pearson correlation analysis was performed between the relative quantification values of 20 key DEMs and various meat quality parameters (Figure 8). The results revealed a tightly interconnected association network between specific DEMs and distinct meat quality attributes. Among the DEMs analyzed, seven exhibited significant correlations with meat color parameters (L*, a*, b*); two were significantly correlated with shear force; three were significantly correlated with cooking loss; eighteen were significantly correlated with pH value; additionally, one DEM showed a significant correlation with moisture content.

Specifically, with respect to meat color and tenderness, cholesteryl laurate exhibited a highly significant positive correlation with shear force (r = 0.52, *p* < 0.01). Regarding water-holding capacity, cooking loss displayed significant positive correlations with three DEMs: anserine, 1-methyl-L-histidine, and 1-(5-phosphoribosyl)imidazole-4-acetate (r = 0.38, 0.42, and 0.38, respectively; *p* < 0.05). In terms of pH value, significant associations were observed with up to 18 DEMs. Among these, anserine, 1-methyl-L-histidine, 1-(5-phosphoribosyl)imidazole-4-acetate, N-acetylhistamine, and hydrocortisone exhibited highly significant positive correlations with pH (r = 0.55, 0.57, 0.69, 0.49, and 0.49, respectively; *p* < 0.01). Conversely, sphingosine-1-phosphate, pantothenic acid, uridine-diphosphate-N-acetylglucosamine, cholesteryl laurate, L-alanine, and D-glutamine displayed highly significant negative correlations with pH (r = −0.58, −0.50, −0.55, −0.55, −0.61, and −0.48, respectively; *p* < 0.01).

Consistent with the proteomic findings, the number of DEMs significantly correlated with pH value was overwhelmingly predominant in the metabolomic dataset, accounting for 18 metabolites. This further corroborates that key metabolites such as anserine and histidine derivatives may serve as essential endogenous buffering systems or metabolic nodes within skeletal muscle, directly participating in the regulation of post-mortem muscle pH homeostasis, thereby systematically influencing meat color stability and water-holding capacity.

### 3.4. Joint Pathway Analysis of Proteomic and Metabolomic Data

**Figure 9:**
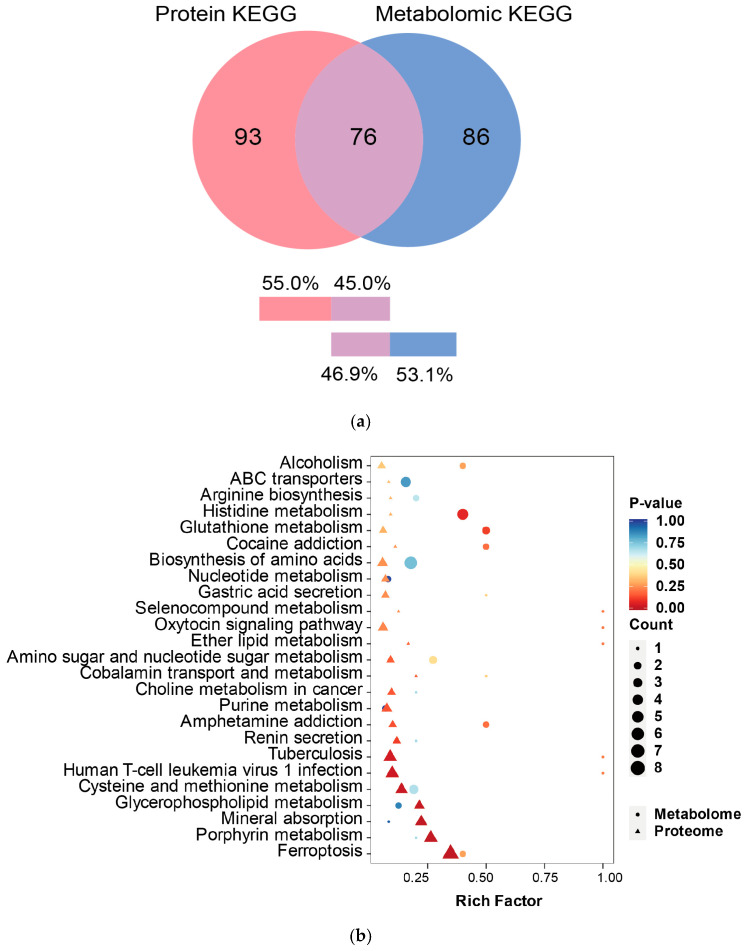

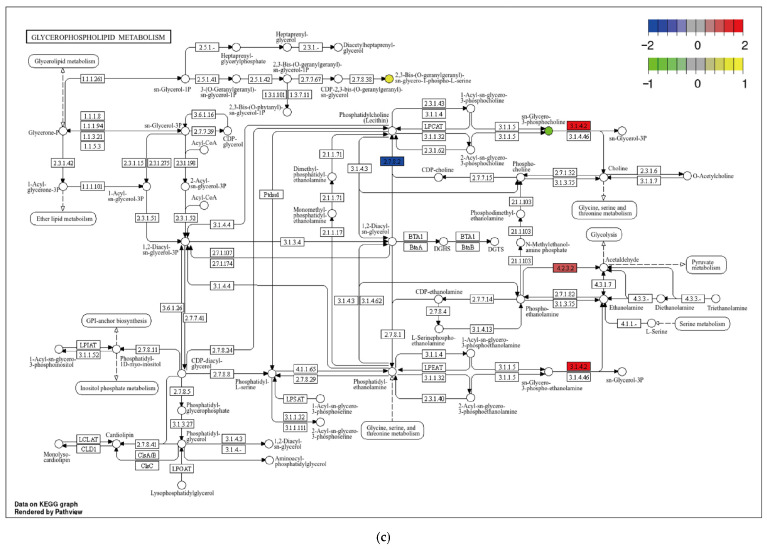
Joint pathway analysis of proteomic and metabolomic data from TBL and ALT sheep (**a**) Venn diagram illustrating the overlap between protein-enriched and metabolite-enriched KEGG pathways. (**b**) Bubble plot of the top 25 co-enriched KEGG pathways ranked by proteomic significance. (**c**) Schematic representation of the glycerophospholipid metabolism pathway. Red indicates upregulated proteins, blue indicates downregulated proteins; yellow indicates upregulated metabolites, and green indicates downregulated metabolites. Solid arrows indicate direct metabolic reactions, while dashed arrows represent indirect links or multi-step reactions within the pathway.

To identify meat quality-associated pathways co-regulated by proteins and metabolites, we performed a co-enrichment analysis of DEPs and DEMs identified between the two breeds. The results revealed that DEPs and DEMs were jointly enriched in 76 KEGG pathways (Figure 9a), thereby establishing a foundation for subsequent identification of core regulatory pathways. To focus on the most critical regulatory networks, the top 25 pathways ranked by proteomic *p*-value are presented (Figure 9b). The results demonstrated significant enrichment in pathways including ferroptosis, porphyrin metabolism, mineral absorption, and glycerophospholipid metabolism. These pathways are broadly implicated in the regulation of muscle nutrient metabolism and internal environmental homeostasis, suggesting that they constitute core pathways governing breed-specific divergence in meat quality. Notably, the glycerophospholipid metabolism pathway, which influences muscle cell membrane composition and lipid metabolism, was subjected to further detailed analysis (Figure 9c). Within this pathway, the key enzymes GPCPD1 and ETNPPL were upregulated, whereas CHPT1 was downregulated. We hypothesize that these three enzymes coordinately regulate glycerophospholipid metabolism, thereby impacting meat quality traits, and thus represent potential molecular targets for subsequent functional validation.

## 4. Discussion

Long-term adaptation of animals to climatic environments is often accompanied by multifaceted physiological remodeling encompassing energy metabolism, muscle development, and stress responses, alterations that may ultimately influence meat quality attributes. Sheep breeds adapted to divergent climatic conditions may have evolved distinct muscle physiological characteristics and meat quality traits. TBL and ALT sheep, as important indigenous breeds from the Turpan and Altay regions of Xinjiang, are adapted to hot and cold climates, respectively, and their meat quality characteristics represent a vital component of germplasm resource value. However, the molecular mechanisms underlying breed-specific divergence in meat quality have yet to be systematically elucidated. In the present study, we employed the *longissimus dorsi* muscle of yearling TBL and ALT rams to conduct comprehensive assessments of meat quality traits, muscle histological characteristics, and joint proteomic and metabolomic analyses. Our findings reveal that the TBL-LD and ALT-LD groups exhibit differences in protein and metabolite composition, muscle fiber architecture, and selected meat quality parameters, which may collectively influence the physicochemical properties and eating quality of post-mortem meat.

pH value is a critical physiological parameter governing post-mortem meat quality, systematically modulating meat color, tenderness, water-holding capacity, and storage stability through its effects on protein conformation, enzymatic activity, and microbial metabolism [17,18]. Cooking loss reflects the water-holding capacity of meat after thermal processing and is inversely correlated with tenderness and juiciness [19,20]. In the present study, the TBL-LD group exhibited significantly lower pH values and cooking loss percentages compared with the ALT-LD group (*p* < 0.01), indicative of superior water-holding capacity. Meat color parameters showed no statistical differences between the two breeds (*p* > 0.05).

Skeletal muscle fiber type composition, diameter, and CSA are known to influence meat quality [21,22]. Although TBL sheep exhibited significantly smaller muscle fiber diameter and CSA, which are traits generally associated with finer texture, their shear force values (60.96 ± 14.77 N) were numerically higher than those of ALT sheep (49.1 ± 20.36 N) (*p* = 0.079). This indicates that TBL meat tends to be tougher. This discrepancy suggests that in these extreme-adapted breeds, tenderness is not solely determined by fiber size but may be heavily influenced by other factors such as intramuscular fat content and connective tissue cross-linking under heat stress. Therefore, each breed presents distinct advantages; TBL excels in water-holding capacity, whereas ALT sheep potentially offer superior tenderness.

Meat serves as an important dietary source of protein and essential minerals for human nutrition. In the present study, crude protein content was numerically higher in TBL lamb than in ALT lamb, whereas crude fat, crude ash, and moisture contents were slightly lower. Although these differences did not attain statistical significance, they are consistent with the known breed characteristics of TBL sheep [11]. Minerals function as structural components or cofactors for proteins and enzymes in animals, participating not only in muscle energy metabolism but also in the regulation of post-mortem meat tenderization [23]. Previous research has demonstrated that meat quality attributes such as lipid peroxidation extent, tenderness, meat color, and juiciness are closely associated with the concentrations of elements including iron, magnesium, and selenium [23,24]. For instance, selenium is an essential constituent of glutathione peroxidase (GPX), thereby enhancing the antioxidant capacity of meat [25,26]. Giro et al. further confirmed that selenium-enriched lamb can alleviate CCl_4_-induced hepatitis in mice while elevating antioxidant status and overall resistance [27]. Magnesium contributes to improved meat color and water-holding capacity by modulating organismal stress responses [28]. In contrast, iron, as the core component of heme, influences both meat color development and flavor stability [29,30]. In the current study, the higher selenium and magnesium contents observed in TBL lamb (Table 2) may potentially contribute to the substrate availability for antioxidant defense systems (such as GPX), although specific enzymatic assays are required to confirm greater functional antioxidant capacity. The lower iron content may reduce the catalytic potential for lipid oxidation, thereby contributing to delayed flavor deterioration. Furthermore, the lower aluminum content mitigates potential risks associated with exogenous element accumulation. Collectively, these differences in mineral element composition highlight distinct potential advantages for TBL lamb with respect to key quality attributes.

Alterations in protein expression profiles reflect, to a considerable extent, the adaptive responses of an organism to environmental stimuli. By comparing the proteomic profiles of the *longissimus dorsi* muscle between TBL and ALT sheep, this study revealed significant inter-breed divergence in multiple metabolic pathways associated with substrate metabolism and cellular homeostasis, primarily encompassing lipid metabolism, carbohydrate metabolism, amino acid metabolism, as well as pathways related to ferroptosis and mineral absorption. Further analysis indicated that the differential expression of key proteins, including GPCPD1, ETNPPL, and FBP1, suggests that TBL sheep may have undergone adaptive adjustments in energy utilization patterns and membrane structural integrity within skeletal muscle through the regulation of glycerophospholipid metabolism and fructose metabolism during long-term adaptation, thereby maintaining normal muscle physiological status.

GPCPD1 (also known as GDE5) is a glycerophosphocholine phosphodiesterase that functions as a key enzyme in the glycerophospholipid metabolism pathway, catalyzing the specific hydrolysis of glycero-3-phosphocholine (GPC) to glycerol-3-phosphate and choline [31]. This protein is specifically expressed in skeletal muscle and is a critical regulator of muscle fiber type specification and muscle development [32,33]. Existing evidence indicates that GPCPD1 function also extends to the modulation of cellular stress responses: transgenic mice with skeletal muscle-specific overexpression of a catalytically inactive GDE5 mutant exhibited pronounced upregulation of heat shock protein HSP70 [34], suggesting that alterations in GPCPD1 expression or activity are intimately linked to stress responses in muscle cells. In the present study, although no marked upregulation of HSP70 was detected in the *longissimus dorsi* muscle of TBL sheep, increased expression of another heat shock protein family member, SERPINH1 (HSP47), was observed. The upregulation of GPCPD1 in TBL sheep under the high-temperature environment of the Turpan region implies that it may mediate molecular adaptation to hot climates through the modulation of myofiber metabolic status. Recent research has demonstrated that GPCPD1 overexpression can drive a shift in skeletal muscle fiber type composition from fast-twitch to slow-twitch fibers [35]. A higher proportion of slow-twitch fibers not only confers greater oxidative metabolic endurance but is also generally regarded as a hallmark of superior meat quality, contributing to enhanced juiciness and flavor [36,37]. Correlation analysis in this study confirmed that GPCPD1 expression levels were significantly negatively correlated with both pH value and cooking loss percentage. Although a lower post-mortem pH is conventionally associated with diminished water-holding capacity, the actually observed lower cooking loss in TBL muscle suggests that GPCPD1-mediated myofiber type conversion (with slow-twitch fibers inherently possessing superior water-holding properties) may exert a compensatory or even predominant role in overall meat quality regulation. This finding provides compelling molecular corroboration for the histological phenotype reported by Nuziguli Tuerxun et al. [38], who observed a significantly greater abundance of type I (slow-twitch) muscle fibers in TBL sheep compared with ALT sheep.

ETNPPL, a rate-limiting enzyme in the glycerophospholipid metabolic pathway, is localized in both the cytoplasm and mitochondria of skeletal muscle. It catalyzes the irreversible cleavage of phosphoethanolamine (PETN), a precursor for the biosynthesis of membrane phospholipids such as phosphatidylethanolamine (PE), yielding acetaldehyde, ammonia, and inorganic phosphate [39]. Upregulation of this enzyme facilitates the alleviation of PETN overaccumulation-induced inhibition of the mitochondrial respiratory chain, thereby improving cellular energy metabolic efficiency [39,40]. Based on these findings, we hypothesize that the elevated expression of ETNPPL in TBL sheep not only contributes to the maintenance of mitochondrial functional homeostasis under heat stress but may also influence the accumulation of volatile flavor precursors through the regulation of phospholipid metabolism and dynamic reorganization of aldehyde species, thereby actively participating in meat quality development.

FBP1 is the core rate-limiting enzyme of the gluconeogenic pathway, specifically catalyzing the hydrolysis of fructose-1,6-bisphosphate (F-1,6-BP) to fructose-6-phosphate (F-6-P) and inorganic phosphate (Pi). Upregulated expression of this enzyme efficiently drives the conversion of non-carbohydrate precursors such as lactate and pyruvate into glucose, thereby augmenting energy reserves and providing substrate support for the elevated energy demands encountered under extreme heat stress conditions [41]. Furthermore, clinical studies have demonstrated that FBP1 can divert glucose carbon flux toward the pentose phosphate pathway (PPP) to maintain NADPH homeostasis, thereby effectively scavenging reactive oxygen species (ROS) accumulation and inhibiting apoptosis [42]. Considering the extremely arid and hot habitat of Turpan Black sheep, we propose that FBP1-mediated reprogramming of glucose metabolism serves not only as a hub for energy remodeling but also as a crucial molecular defense mechanism that reinforces cellular redox homeostasis and confers resistance against heat-induced oxidative stress.

With respect to mineral metabolism, the present study observed significant upregulation of transferrin receptor (TFRC), which is involved in iron uptake, coupled with significant downregulation of ferritin heavy chain 1 (FTH1), responsible for iron storage, in the *longissimus dorsi* muscle of TBL sheep. This expression pattern suggests that TBL sheep adopt an adaptive strategy in mineral metabolism characterized by enhanced uptake and reduced storage, leading to elevated levels of free iron to preferentially meet the substrate demands of high oxygen-consumption metabolic pathways such as mitochondrial oxidative phosphorylation [43,44]. This mechanism elegantly accounts for the phenotypic observation in our meat quality assessment that total iron content was significantly lower in TBL muscle than in ALT muscle. During adaptation to hot environments, muscle cells of TBL sheep appear to rapidly channel absorbed iron into functional metabolic cascades rather than depositing it in inert forms to cope with heightened metabolic and oxidative stress. Concurrently, the significantly higher selenium and magnesium concentrations in TBL muscle relative to ALT muscle further reinforce the foundation of their environmental adaptation. The former, as an essential constituent of glutathione peroxidase, strengthens the antioxidant defense system, whereas the latter, as a critical cofactor for ATP-related enzymes, safeguards energy metabolic homeostasis under high-temperature conditions [25,28].

In the present study, non-targeted metabolomic analysis was employed to systematically characterize the substantial differences in the metabolic profiles of the *longissimus dorsi* muscle between TBL and ALT sheep. Pathway enrichment analysis revealed that the Apelin signaling pathway and the phospholipase D (PLD) signaling pathway constitute key metabolic hubs driving breed-specific divergence in meat quality traits. Notably, sphingosine-1-phosphate (S1P) and cAMP, serving as core regulatory nodes, exhibited a coordinated upregulation trend in both pathways; concurrently, L-glutamate displayed pronounced and specific accumulation within amino acid and PLD metabolic networks. These metabolic phenotypes provide direct evidence for elucidating the high-temperature environmental adaptation of TBL sheep and the mechanisms underlying their distinctive meat quality characteristics.

At the level of lipid metabolism, although a significant upregulation of the conventional pro-lipolytic signaling molecule cAMP was detected in TBL muscle, we postulate that the associated pathways may be subject to dominant suppression by other regulatory factors. Previous studies have demonstrated that Apelin peptides can potently inhibit lipolysis through dual molecular mechanisms: on the one hand, Apelin activates AMP-activated protein kinase (AMPK) via Gq protein, which catalyzes the phosphorylation of hormone-sensitive lipase (HSL) at the Ser-565 residue, thereby inhibiting its enzymatic activity; on the other hand, Apelin can suppress adenylate cyclase activity through Gi protein, reducing protein kinase A (PKA) activity and consequently diminishing phosphorylation at the activating Ser-563 site of HSL [45]. These two parallel pathways collectively achieve effective blockade of lipolysis. Based on this evidence, we hypothesize that in TBL sheep, compensatory activation of the Apelin signaling pathway may effectively counteract the pro-lipolytic effect induced by cAMP accumulation, ultimately resulting in a net suppression of lipid degradation within skeletal muscle. Concurrently, the upregulation of S1P, which parallels that of cAMP, further reinforces this metabolic reprogramming profile. Published evidence confirms that S1P inhibits preadipocyte differentiation in a dose-dependent manner, with mechanisms involving not only the downregulation of the key adipogenic transcription factor PPARγ but also the broad suppression of MAPK signaling cascades including ERK, p38, and JNK [46], which exhibits ideal synergistic complementarity with Apelin-mediated suppression of the MAPK/ERK adipogenic pathway [47]. Accordingly, we propose a novel hypothesis: to cope with the extreme hot environment, TBL sheep have evolved a survival strategy that coordinately activates the Apelin and S1P signaling networks to exert bidirectional suppression of both adipogenesis and lipolysis within skeletal muscle, aimed at minimizing overall lipid turnover rates and metabolic heat production. This distinctive reconfiguration of lipid metabolic homeostasis would inevitably alter intramuscular fat deposition patterns and fatty acid composition, thereby profoundly influencing the tenderness, juiciness, and overall sensory attributes of lamb from this breed.

Beyond the adaptive remodeling of lipid metabolism, the specific enrichment of amino acid metabolism also constitutes a fundamental basis for the meat quality characteristics of TBL sheep. Metabolomic data revealed a highly significant accumulation of L-glutamate in TBL muscle, which was extensively involved in 16 core pathways, including protein digestion and absorption and amino acid biosynthesis. L-glutamate serves not only as a pivotal nodal substrate for protein synthesis and energy metabolism but also as the foremost amino acid contributing to the umami taste of meat [48]. Its substantial enrichment in TBL muscle tissue provides direct metabolomic corroboration of the organoleptic consensus that TBL lamb possesses a rich flavor profile with pronounced umami characteristics, and it may be regarded as a signature metabolite distinguishing the flavor profile of TBL sheep from that of ALT sheep.

Collectively, the multi-omics data generated in this study have effectively identified key metabolic pathways and molecular clusters governing meat quality and environmental adaptation. Nevertheless, it should be noted that metabolomic profiling primarily captures a static concentration snapshot of metabolites at a given time point, whereas metabolic networks in vivo are highly dynamic. The complex, and occasionally seemingly antagonistic, functions exhibited by molecules such as S1P and cAMP in lipid metabolic regulation ultimately depend on the specific tissue microenvironment, cell type, and upstream/downstream interaction networks. Future investigations employing advanced techniques such as isotope-labeled metabolic flux analysis are warranted to further delineate the spatiotemporal dynamics of these key metabolites in the formation of meat quality traits.

By conducting joint analyses of proteomic and non-targeted metabolomic data, this study systematically compared the molecular network characteristics of the *longissimus dorsi* muscle between the heat-adapted TBL sheep and the cold-adapted ALT sheep. Joint pathway enrichment analysis revealed that glycerophospholipid metabolism, cysteine and methionine metabolism, and the ferroptosis pathway constitute the core differential pathways driving breed divergence (Figure 9b). The interplay and synergy among these three major metabolic modules form the critical molecular foundation underlying the adaptation of TBL sheep to extreme heat and the concomitant remodeling of muscle quality.

Under heat stress conditions, the biophysical properties of cellular membranes are highly susceptible to damage, which can subsequently trigger irreversible cellular injury. Existing evidence demonstrates that glycerophospholipid metabolism serves as a central pathway in response to heat stress, achieving dynamic membrane lipid remodeling through the regulation of the biosynthesis and stoichiometric ratio of core membrane constituents phosphatidylcholine (PC) and phosphatidylethanolamine (PE), thereby effectively mitigating heat-induced membrane damage [49,50]. The proteomic data in this study accurately captured active signals within the glycerophospholipid metabolic pathway in TBL sheep: not only were the catabolic enzymes GPCPD1 and ETNPPL significantly upregulated, but the expression of CHPT1, a key enzyme in PC synthesis, was downregulated. Concurrently, metabolomic data provided cross-validation, revealing significantly reduced levels of the substrate GPC. Mechanistically, the upregulation of GPCPD1 accelerates GPC hydrolysis, thereby directly limiting PC synthesis via the CDP-choline pathway [31,51], whereas the enhanced activity of ETNPPL promotes the catabolism of PE precursors [52]. This series of coordinated multi-omics alterations strongly suggests that TBL sheep initiate a profound remodeling of myocellular glycerophospholipid metabolism under heat stress. Notably, GPC itself is widely recognized as a highly effective organic osmoprotectant, possessing crucial functions in stabilizing cell membrane structure and maintaining cellular volume [53,54]. Therefore, the significant reduction in muscle GPC levels observed in TBL sheep essentially reflects a compensatory process wherein this molecule is mobilized and turned over at an accelerated rate to participate in osmoregulation under sustained heat stress. This micro-level osmotic homeostatic regulation aligns perfectly with the macro-level phenotypic observation that TBL sheep exhibit a significantly lower cooking loss percentage than ALT sheep, thereby providing a molecular annotation for their superior muscle water-holding capacity.

With respect to the antioxidant defense system, adaptive alterations in the cysteine and methionine metabolic pathway delineate a distinctive redox homeostasis strategy in TBL sheep. Multi-omics data revealed that, in TBL sheep, this pathway is characterized by the upregulation of PSAT1 and AHCYL2 proteins and the downregulation of MTR and APIP. At the metabolomic level, this was mirrored by the consumption-associated downregulation of L-cysteine (Cys) and the substantial accumulation of L-alanine (Ala), homocysteine (Hcy), and L-homoserine (Hse). This metabolic signature implies that the muscle tissue of TBL sheep is subjected to a higher intensity of oxidative stress. As the key rate-limiting precursor for the synthesis of the core antioxidant glutathione (GSH), the pronounced depletion of Cys directly reflects the markedly increased antioxidant burden imposed by the clearance of heat-induced ROS [55]. Concomitantly, the upregulation of PSAT1 may compensatorily promote the generation of serine (Ser) and glycine (Gly), thereby providing a sustained supply of substrates for de novo GSH biosynthesis [56]. Furthermore, the remodeling of the sulfur-containing amino acid metabolic network not only establishes a cornerstone for antioxidant defense, but the accumulation of metabolic intermediates also furnishes abundant precursors for the generation of characteristic sulfur-containing volatile flavor compounds in lamb, thereby profoundly influencing the unique flavor profile of TBL meat [57,58].

More critically, the aforementioned metabolic pathway alterations ultimately converge functionally within the ferroptosis regulatory network. Ferroptosis is an iron-dependent form of programmed cell death driven by lipid peroxidation [59,60]. In heat stress models, the system typically becomes highly susceptible to ferroptosis due to iron homeostasis imbalance, rampant ROS generation, and the collapse of the GSH-GPX4 antioxidant axis. Once initiated, this process directly compromises myocellular membrane integrity, leading to a precipitous decline in muscle water-holding capacity, a surge in cooking loss, and overall meat quality deterioration [61,62]. In the present study, TBL sheep exhibited upregulation of TFRC and downregulation of FTH1. This aggressive iron metabolic strategy, adopted to meet the elevated energy demands under high ambient temperatures, objectively increases the potential risk of triggering ferroptosis. However, TBL sheep have exquisitely evolved to maintain bilayer stability of membrane lipids through the aforementioned remodeling of glycerophospholipid metabolism and to robustly preserve the functionality of the GSH-GPX4 axis via antioxidant adaptation in cysteine metabolism, thereby successfully antagonizing the onset of ferroptosis under extreme metabolic stress.

In summary, through in-depth joint pathway analysis of proteomic and metabolomic data, this study elucidates the molecular divergence between TBL and ALT sheep in the pathways of glycerophospholipid metabolism, cysteine and methionine metabolism, and ferroptosis. These highly interactive metabolic networks collectively not only constitute the physiological defense barrier enabling TBL sheep to withstand extreme thermal environments but also, through the regulation of cellular water-holding capacity and antioxidant function, ultimately manifest as specific meat quality phenotypes, including reduced cooking loss and distinctive flavor characteristics. This study provides a novel multi-omics perspective for systematically deciphering the coupling mechanisms between climatic adaptation and meat quality formation in sheep and other ruminants.

## 5. Conclusions

This study aimed to elucidate the molecular mechanisms underlying the divergence in meat quality between two indigenous sheep breeds adapted to extreme climates. We demonstrate that heat-adapted TBL sheep and cold-adapted Altay sheep exhibit distinctly divergent meat quality phenotypes, with TBL primarily characterized by a finer muscle fiber structure, enhanced water-holding capacity, and specific mineral enrichment. Through joint pathway analysis of proteomic and non-targeted metabolomic data, we identified 99 key differentially expressed proteins and 364 differential metabolites. The core molecular divergence between the two breeds was predominantly concentrated in lipid and amino acid metabolism networks, specifically the glycerophospholipid metabolism and ferroptosis pathways. Crucially, within the lipid remodeling process, the metabolite GPC was identified as a central regulatory node. Through synergistic interactions with key enzymes such as GPCPD1 and ETNPPL, GPC profoundly influences muscle cell membrane homeostasis and water-holding capacity.

In summary, this study comprehensively delineates the multi-omics molecular networks driving meat quality divergence and provides profound insights into the biological coupling between extreme climatic adaptation and meat quality formation. These findings offer a robust theoretical foundation for future molecular-assisted breeding of stress-resilient, high-quality meat sheep.

## Figures and Tables

**Table 1:** Differences in the meat quality physical indexes between Turpan Black sheep and Altay sheep.

Index	Turpan Black Sheep	Altay Sheep	*p*-Value
Live weight (kg)	32.65 ± 3.45	32.36 ± 3.52	0.820
Carcass weight (kg)	14.27 ± 1.95	14.42 ± 1.97	0.836
Slaughter rate (%)	43.84 ± 2.02	44.60 ± 4.30	0.552
pH	6.85 ± 0.02	6.99 ± 0.04	<0.001 **
L*	33.55 ± 5.29	32.65 ± 3.16	0.576
a*	18.16 ± 2.48	18.52 ± 2.99	0.722
b*	20.52 ± 1.93	21.4 ± 3.13	0.368
Muscle shear force (N)	60.96 ± 14.77	49.1 ± 20.36	0.079
Cooking loss (%)	28.00 ± 5.00	35.00 ± 6.00	0.003 **

Note: Values are presented as mean ± standard deviation (SD). ** *p* < 0.01. The same notation applies hereinafter.

**Table 2:** Differences in routine nutritional components and mineral content between Turpan Black sheep and Altay sheep.

Index	Turpan Black Sheep	Altay Sheep	*p*-Value
Crude protein content (%)	21.90 ± 1.26	21.87 ± 1.34	0.961
Crude fat content (%)	0.98 ± 0.60	1.16 ± 0.58	0.413
Moisture content (%)	76.94 ± 0.75	77.13 ± 0.80	0.507
Crude ash content (%)	1.23 ± 0.17	1.33 ± 0.14	0.096
Al (mg/kg)	1.50 ± 1.26	2.21 ± 1.22	0.028 *
Cu (mg/kg)	0.89 ± 0.32	0.93 ± 0.17	0.516
Zn (mg/kg)	23.37 ± 3.51	25.02 ± 5.28	0.160
Se (mg/kg)	0.09 ± 0.01	0.03 ± 0.01	0.000 **
Fe (mg/kg)	15.89 ± 2.14	17.37 ± 1.87	0.006 **
Ca (mg/kg)	36.37 ± 3.18	38.15 ± 4.22	0.069
Mg (mg/kg)	250.30 ± 23.46	237.63 ± 15.97	0.018 *
Na (mg/kg)	537.90 ± 66.23	542.17 ± 54.23	0.786

Note: Values are presented as mean ± standard deviation (SD). * *p* < 0.05; ** *p* < 0.01.

## Data Availability

The data presented in this study are openly available in [FigShare, at http://doi.org/10.6084/m9.figshare.29551862].

## Abbreviations

The following abbreviations are used in this manuscript:

TBL	Turpan Black
CSA	cross-sectional area
HE	eosin
DTT	dithiothreitol
IAA	iodoacetamide
TFA	trifluoroacetic acid
DIA	data-independent acquisition
HCD	Higher-energy collisional dissociation
MBR	Match-between-runs
FDR	false discovery rate
ESI	electrospray ionization
Arb	arbitrary units
cps	counts per second
KNN	k-nearest neighbor
SVR	support vector regression
CV	coefficient of variation
QC	quality control
DEPs	differentially expressed proteins
DEMs	differential metabolites
VIP	variable importance in projection
OPLS-DA	orthogonal partial least squares-discriminant analysis
GO	Gene Ontology
KEGG	Kyoto Encyclopedia of Genes and Genomes
R^2^Y	explanatory power
Q^2^	predictive capability
BP	biological process
CC	cellular component
MF	molecular function
LTF	lactotransferrin
PSAT1	phosphoserine aminotransferase 1
FBP1	fructose-1,6-bisphosphatase 1
CP	ceruloplasmin
PM20D1	N-fatty-acyl-amino acid synthase/hydrolase
LC–MS/MS	liquid chromatography–tandem mass spectrometry
cAMP	cyclic adenosine monophosphate
GPX	glutathione peroxidase
GPC	the specific hydrolysis of glycero-3-phosphocholine
PETN	phosphoethanolamine
F-1,6-BP	fructose-1,6-bisphosphate
F-6-P	fructose-6-phosphate
Pi	inorganic phosphate
PPP	pentose phosphate pathway
ROS	reactive oxygen species
TFRC	transferrin receptor
FTH1	ferritin heavy chain 1
PLD	phospholipase D
S1P	sphingosine-1-phosphate
AMPK	Apelin activates AMP-activated protein kinase
HSL	hormone-sensitive lipase
PKA	protein kinase A
PC	phosphatidylcholine
PE	phosphatidylethanolamine
Cys	L-cysteine
Ala	L-alanine
Hcy	homocysteine
Hse	L-homoserine
GSH	glutathione
Ser	serine
Gly	and glycine
